# Costs and Causes of Oncology Drug Attrition With the Example of Insulin-Like Growth Factor-1 Receptor Inhibitors

**DOI:** 10.1001/jamanetworkopen.2023.24977

**Published:** 2023-07-28

**Authors:** Valerie Jentzsch, Leeza Osipenko, Jack W. Scannell, John A. Hickman

**Affiliations:** 1 London School of Economics, London, United Kingdom; 2 Consilium Scientific, London, United Kingdom; 3 Science, Technology, and Innovation Studies, University of Edinburgh, Edinburgh, United Kingdom; 4 JW Scannell Analytics LTD, Edinburgh, United Kingdom; 5 School of Biological Sciences, University of Manchester, Manchester, United Kingdom

## Abstract

**Importance:**

The development of oncology drugs is expensive and beset by a high attrition rate. Analysis of the costs and causes of translational failure may help to reduce attrition and permit the more appropriate use of resources to reduce mortality from cancer.

**Objective:**

To analyze the causes of failure and expenses incurred in clinical trials of novel oncology drugs, with the example of insulin-like growth factor-1 receptor (IGF-1R) inhibitors, none of which was approved for use in oncology practice.

**Design, Setting, and Participants:**

In this cross-sectional study, inhibitors of the IGF-1R and their clinical trials for use in oncology practice between January 1, 2000, and July 31, 2021, were identified by searching PubMed and ClinicalTrials.gov. A proprietary commercial database was interrogated to provide expenses incurred in these trials. If data were not available, estimates were made of expenses using mean values from the proprietary database. A search revealed studies of the effects of IGF-1R inhibitors in preclinical in vivo assays, permitting calculation of the percentage of tumor growth inhibition. Archival data on the clinical trials of IGF-1R inhibitors and proprietary estimates of their expenses were examined, together with an analysis of preclinical data on IGF-1R inhibitors obtained from the published literature.

**Main Outcomes and Measures:**

Expenses associated with research and development of IGF-1R inhibitors.

**Results:**

Sixteen inhibitors of IGF-1R studied in 183 clinical trials were found. None of the trials, in a wide range of tumor types, showed efficacy permitting drug approval. More than 12 000 patients entered trials of IGF-1R inhibitors in oncology indications in 2003 to 2021. These trials incurred aggregate research and development expenses estimated at between $1.6 billion and $2.3 billion. Analysis of the results of preclinical in vivo assays of IGF-1R inhibitors that supported subsequent clinical investigations showed mixed activity and protocols that poorly reflected the treatment of advanced metastatic tumors in humans.

**Conclusions and Relevance:**

Failed drug development in oncology incurs substantial expense. At an industry level, an estimated $50 billion to $60 billion is spent annually on failed oncology trials. Improved target validation and more appropriate preclinical models are required to reduce attrition, with more attention to decision-making before launching clinical trials. A more appropriate use of resources may better reduce cancer mortality.

## Key Points

**Question:**

What are the associated expenses of clinical research and what factors underly the translational failure of inhibitors of the insulin-like growth factor-1 receptor (IGF-1R) in oncology?

**Findings:**

In this cross-sectional study, 16 inhibitors of IGF-1R underwent 183 clinical trials in more than 12 000 patients; none of the agents was approved for clinical use in oncology practice and the trials were estimated to have had expenses of greater than $1.6 billion. Half of the published in vivo preclinical data analyzed showed less than a 50% inhibition of tumor growth by IGF-1R inhibitors.

**Meaning:**

With high attrition rates for oncology drugs, the fruitless and expensive clinical trials of 16 IGF-1R inhibitors draw attention to the need for improved preclinical models and better decision-making before trials are launched, reducing substantial financial losses and avoiding exposure of patients to potential toxic effects.

## Teaser

This cross-sectional study examines the costs generated in clinical trials, using the research and development studies of 16 insulin-like receptor-1 inhibitors in cancer, none of which was approved for use in the oncology practice setting, as an example.

## Introduction

Over the past 2 decades, the pharmaceutical industry has become heavily focused on the discovery of therapeutic agents for the treatment of metastatic cancer.^1^ These agents largely either target genetic changes implicated in cancer pathology to provide targeted therapies^2^ or they modulate the immune system.^3^ However, development of oncology drugs has been reported to have a persistent attrition rate of greater than 95%,^4,5,6^ highlighting the disparity between positively assessed preclinical drug activity and subsequent inactivity in patients. The costs of clinical trials in oncology have been estimated to exceed those of other therapeutic areas so that failure is likely to be expensive.^7^ Failure is not only expensive in time and money but is disappointing for the scientists and clinicians dedicated to projects that flounder. Failure is ultimately most disappointing for patients, some of whom may be exposed in clinical trials to therapeutically inactive drugs that carry toxicity.

To our knowledge, estimates of the costs of clinical drug attrition in oncology have not been published. We have chosen to estimate the development expenses associated with the search for clinically active, targeted inhibitors of the insulin-like growth factor-1 receptor (IGF-1R).^8,9,10,11,12^ Insulin-like growth factor-1 receptor inhibitors (ie, small molecules, antibodies, and cell therapy), were chosen as an example as we were able to capture the results for 16 IGF-1R inhibitors evaluated in trials against a broad range of tumor types by different pharmaceutical and biotechnology companies, many of which had published preclinical data. All the inhibitors, alone or in drug combinations, failed to demonstrate clinical activity deemed sufficient for approval in oncology practice.

We have estimated the expense of the clinical phases of IGF-1R inhibitor development programs, collating details of treatments, including the numbers of patients entered in trials. We discuss the quality of the preclinical data leading to the launch of IGF-1R inhibitors into clinical trials and address more broadly the financial consequences of failure. We attempt to place the failure of the IGF-1R inhibitor programs into a temporal scientific and clinical context.

## Methods

### Creation of Databases of Clinical Trials of IGF-1R Inhibitors

This study of published research data did not involve personal medical records and does not constitute human participant research. We searched public databases (Google Scholar, PubMed, SCOPUS, and Web of Science) to find details on inhibitors of IGF-1R. Specifically, the key words used were *molecular IGF-1R*, therap*, targeted therap*, *cancer*, *oncology*, *clinical trial*, and *drug development*.

A database of clinical trials of IGF-1R inhibitors was then created by searching the ClinicalTrials.gov registry (NCT)^13^ for the key terms IGF-1R and the individual drug names or codes, with the date range from January 1, 2000, to July 31, 2021. Clinical trials not in the field of oncology, identified by pathology, were removed from the database. Trials were annotated for trial title, drug code, trial sponsor (industry or other), cancer indication, date of trial, patient numbers, trial phase, and program costs. Consideration of the study lead organizations allowed an insight into the primary sources of financing for each clinical trial. As such, organizations were considered to belong to the pharmaceutical or biotechnology industry, academia, the US National Cancer Institute, or other.

### Estimate of Patient Numbers

We estimated patient numbers from reports in the NCT database^13^ for the key terms IGF-1R and the individual drug names or codes, again with the date range January 1, 2000, to July 31, 2021. Where patient numbers were not reported (14 of 183 entries), we assumed that the trial had recruited the same number of patients as the mean of other IGF-1R trials of the same phase.

### Estimate of Clinical Development Expenses

The first research and development (R&D) expense estimates were from a proprietary database made accessible to us by Evaluate Ltd,^14^ which provided data for 129 of the total of 183 trials found. United States publicly traded companies are required by the Securities and Exchange Commission to file a detailed annual report, known as a 10-K. For many small and medium-sized biotechnology and pharmaceutical companies, the 10-K, and sometimes other publicly available documents, contains R&D expense information at the level of individual drugs. Evaluate Ltd collates this drug-level expense information on a nominal basis (ie, not inflated or deflated to a reference year) and combines it with other data in the public domain (eg, from ClinicalTrials.gov and other company disclosures in the 10-K) to derive per-patient benchmarks. The empirical phase 2 and 3 trial benchmarks consider a fixed trial expense plus a per-patient expense. There are further adjustments for geographic location and trial duration vs the technology-by-EPHMRA benchmark. The benchmark estimates are checked by comparing forecast R&D expenses with company-reported R&D expenses for the top 20 global biopharmaceutical firms. Evaluate Ltd method centers on what can be thought of as an accounting view of R&D expenses since they are derived from accounting-based reports (eg, 10-K filings). The Evaluate Ltd figures do not include the cost of capital that one would see in an investment view of R&D costs. However, the figures in the 10-K filings will generally include program expenses above the narrow cost of clinical trials (eg, manufacture of drugs for trials, any ongoing nonhuman toxicology studies, data processing, and preparing for regulatory submissions). Furthermore, companies will vary in what they exclude and include in when they report drug-level R&D expenses in their 10-Ks. We note that the estimates of Evaluate Ltd are used extensively in the drug industry by companies that know their own R&D costs. Details and a general primer on R&D cost estimates are provided in the eMethods in Supplement 1.

### Estimate of Expenditure

We found 54 clinical trials of IGF-1R inhibitors for which Evaluate Ltd did not provide an expense estimate. To estimate these expenses, the mean cost per patient per trial phase was calculated from the Evaluate Ltd data given in eTable 1 in Supplement 2 and eTable 3 in Supplement 2) and multiplied by the number of patients described in the NCT database^13^ for each of these 54 trials. Eleven of these trials were performed by industry or biotechnology companies and we assumed their costs at the mean (per phase) of trial expenses estimated by Evaluate Ltd. Since estimates of the costs of academic and other trials, such as those by the US National Cancer Institute, are inaccessible, we estimated a range of possible expenses at 1 × the Evaluate Ltd mean, 0.5 × the Evaluate Ltd mean, and 0.2 × the Evaluate Ltd mean (eMethods in Supplement 1).

### Basket Trials

When the IGF-1R inhibitors were in used in basket trials (ie, IGF-1R inhibitors were only one of several different interventions), we first took the expense of the complete basket trial and divided this by the total number of patients enrolled to determine the expense per patient. We then investigated the numbers of patients enrolled in the IGF-1R inhibitor arm from available publications and estimated the expense of that arm alone by multiplying the cost per patient by the number of patients in the IGF-1R inhibitor arm.

### Analysis of Selected In Vivo Preclinical Data

A search for published articles describing preclinical data in vivo for each inhibitor was made using PubMed, using the code number or drug name reported in Table 1 (with one drug for oncology only, subsequently developed for Graves disease by Horizon^15^). The percentage tumor growth inhibition was calculated as described in Carboni et al^16^ by using values shown in the published graphic representations of the results of in vivo assays of the effects of single IGF-1R inhibitors.

**Table 1.** The 16 IGF-1R Inhibitors and the Estimated Number of Patients Entered Into Clinical Trials^a^

Drug name	IGF-1R inhibitor type	Company	Estimated No. of patients
AMG479 (ganitumab)	Antibody	Amgen/NantCell	2864
AVE1642	Antibody	Sanofi-Aventis	57
AXL1717	Small molecule	Axelar AB	204
BIIB022	Antibody	Biogen Idec	98
BMS-754807	Small molecule	Bristol-Myers Squibb	296
CP-751 871 (figitumumab)	Antibody	Pfizer	2029
IGV-001	Antisense/cell therapy	Imvax	93
IMCA12 (cixutumumab)	Antibody	Eli Lilly and Company/NCI	2791
KW-2450	Small molecule	Kyowa Hakko Kirin Pharma Inc	83
MK7454 (robatumumab)	Antibody	Merck & Co/Schering Plough	305
MK0646 (dalotuzumab)	Antibody	Merck, Sharpe & Dohme Corp	1436
MM141 (istiratumab)	Antibody	Merrimack Pharmaceuticals	135
OSI906 (linsitinib)	Small molecule	Oncogene Sciences/Astellas Pharma Inc	1277
PL225B	Small molecule	Piramal Enterprises Ltd	70
RG1507 (teprotumumab)	Antibody	Hoffmann-La Roche	525^b^
XL228	Small molecule	Exelixis	133

Abbreviations: IGF-1R, insulin-like growth factor-1 receptor; NCI, National Cancer Institute.

^a^ The estimated number of patients in trial programs with data taken from eTables 1 and 2 in Supplement 2.

^b^ Oncology only, subsequently developed for Graves disease by Horizon.^15^

## Results

### Clinical Trial Data and Program Expenditures

We found 16 IGF-1R inhibitors, small molecules, a mixed antisense cell therapy, and antibodies that entered oncology clinical trials between 2003 and 2021 (Table 1). Table 1 also presents our estimate of the number of patients who were entered into trial programs as found in the NCT database^13^ and, if not, the mean patient number was used for each phase. We estimate that a total of 12 396 patients were entered into 183 trial programs. Details are presented in eTable 1 and eTable 2 in Supplement 2, with basket trials reported separately.

**Figure 1.**
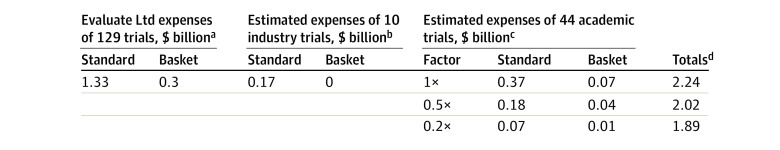
Estimates of Expenses for the 183 IGF-1R Inhibitor Programs IGF-1R indicates insulin-like growth factor-1 receptor. ^a^Expenses incurred provided by Evaluate Ltd for 129 programs performed by industry and biotechnology companies. ^b^Estimated expenses of 10 industry programs not provided by Evaluate Ltd (Methods section). ^c^Estimated expenses of 44 nonindustry and biotechnology programs not provided by Evaluate Ltd (Methods section; eTables 1-3 in Supplement 2). ^d^Estimates of expenses for 183 IGF-1R programs.

Figure 1 presents the estimated R&D expenses of the 129 clinical trials that were costed by Evaluate Ltd ($1.63 billion; eTable 1 in Supplement 2) for trials initiated by industry. It also reports our estimates (eTable 2 and eTable 3 in Supplement 2 and the eMethods in Supplement 1) for the remaining 54 trials not costed by Evaluate Ltd using factors explained in the Methods section to estimate expenditure.

**Figure 2.**
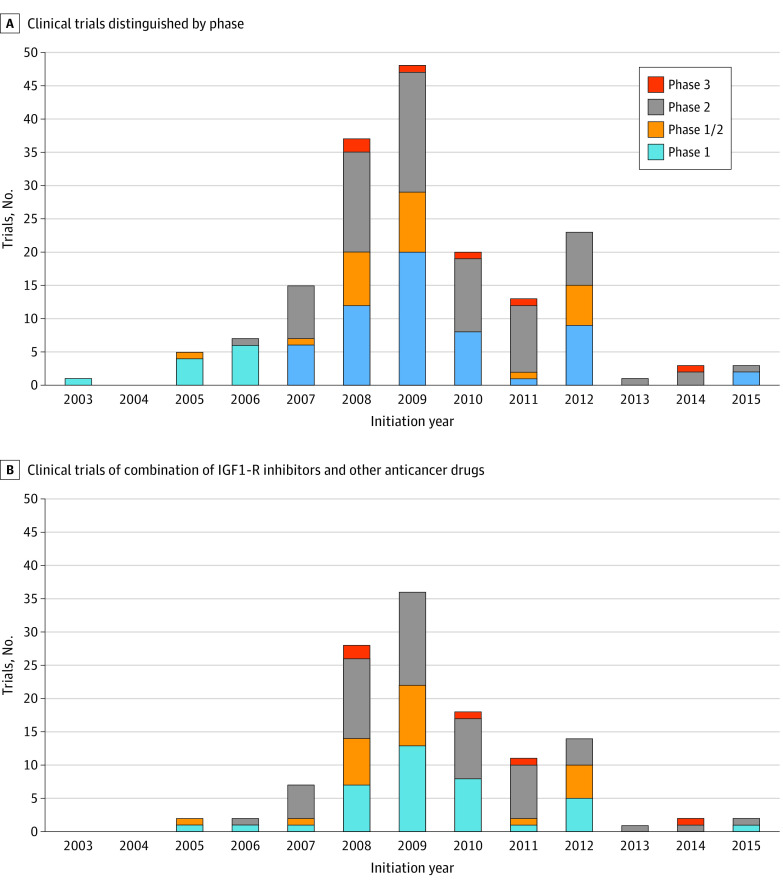
Numbers of Clinical Trials of Insulin-Like Growth Factor-1 Receptor (IGF-1R) Inhibitors A, Number of clinical trials of IGF-1R inhibitors distinguished by phase, initiated in the period 2003 to 2015. B, Number of clinical trials started using combinations of an IGF-1R inhibitor with other anticancer drugs. Details on these trials are reported in eTables 1 and 2 in Supplement 2.

**Figure 3.**
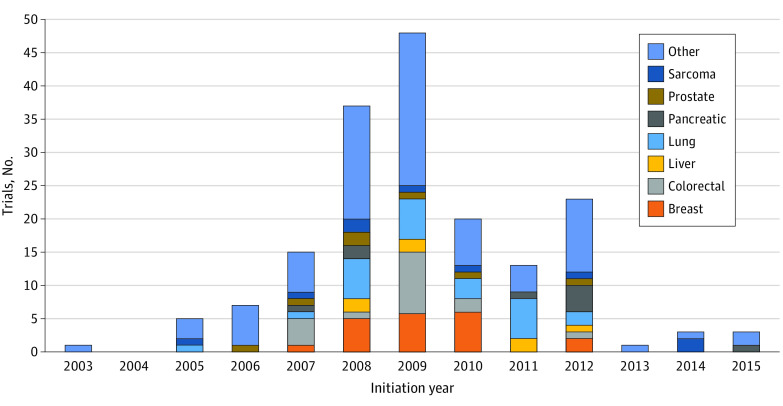
The Principal Cancers Under Investigation in Clinical Trials of Insulin-like Growth Factor-1 Receptor Inhibitors From 2003 to 2021 Specification of the category of others is included in eTables 1 and 2 in Supplement 2 included adrenocortical tumors, brain tumors, carcinomas (not specified), chronic myeloid leukemia, gastroesophageal tumors, head and neck tumors, melanoma, mesothelioma, multiple indications, multiple myeloma, neoplasms (not specified), neuroendocrine, nonhematologic, ovarian, solid tumors (not specified), thymus, and not specified.

Figure 2A shows the number of clinical trials by trial phase (1-3) that were initiated from 2000 to 2015. The first clinical phase 1 trials, using a single IGF-1R inhibitor figitumumab, began in 2003, with phase 3 trials starting in 2008. The number of trials initiated each year increased from 2003 to 2012 and then decreased substantially from 2013 onward. The data for Figure 2A are taken from eTables 1 and 2 in Supplement 2 and are cutoff at 2015 for clarity. Figure 2B shows, for each year, the number of clinical trials using combinations of an IGF-1R inhibitor with other anticancer drugs, broken down by trial phase. Details of these combinations can be found in eTables 1 and 2 in Supplement 2. Figure 3 shows, year by year, trials in which the cancer (eg, breast, colon, and prostate) being treated could be extracted from publicly available data or the proprietary data from the Evaluate Ltd and our database eTables 1 and 2 in Supplement 2).

### Analysis of a Selection of In Vivo Preclinical Data

Table 2 presents a survey of the published preclinical in vivo data testing IGF-1R inhibitors, detailing the inhibitor used, the publication details, the xenografted tumor type, brief observations on the assay methods, and results, with estimates of percentage tumor growth inhibition for 9 inhibitors tested as a single agent against a broad range of tumor xenografts.^16,17,18,19,20,21,22,23,24,25,26,27,28,29,30,31,32,33,34,35,36,37,38,39,40,41,42,43,44,45,46,47,48,49,50^ Of the 62 cell line results annotated, 31 had a percentage of tumor growth inhibition of less than 50%.

**Table 2.** Activity of Single Agent IGF-1R Inhibitors on a Selection of Tumor Xenograft Models

Drug code (name) and source	Tumor type^a^	End point	Comments	Cell line and estimated %TGI^b^
**AMG479 (ganitumab)**				
Fahrenholtz et al,^17^ 2013	Prostate (VACaP)	Regression and cytostasis	Twice weekly for 3 wk; tumor regrowth following cessation of treatment	VACaP, 92%
Beltran et al,^18^ 2009	Pancreatic (BxPC3 and MiaCaPa2)	Growth delay	Palpable tumors remaining	BxPCR, 82%; MiaCaPa2, 83%
Beltran et al,^19^ 2014	Ovarian (OV90, OVCAR3, and TOV −21G)	Stasis (OV90); growth delay (OVCAR3); no effect (TOV-21G)	Twice weekly to end point	OV90, 100%; OVCAR3 57%; TOV-21G, 0%
Tabernero et al,^20^ 2015	Colon (colon-205)	Modest growth delay, leaving palpable tumors	Twice weekly to end point (3 wk)	Colon-205, 35%
Beltran et al,^21^ 2011	Ewing sarcoma (SK-ES-1 and A673) and osteosarcoma (SJSA-1)	Growth delay leaving palpable tumors	Twice weekly to end point (3 wk)	SK-ES-1, 50%; A673, 43%; SJSA-1, 33%
**AVE1642**				
Geoerger et al,^22^ 2010	Neuroblastoma (IGR-N91 and SK-N-AS)	Modest growth delay leaving palpable tumors	Twice weekly to end point (21 or 28 d)	IGR-N91, 22%; SKN-AS, 19%
**BMS-754807**				
Litzenburger et al,^23^ 2011	Triple negative breast cancer tumor graft	Growth delay leaving palpable tumors	Daily until end point (28 d)	61%
Kolb et al,^24^ 2011	Pediatric tumors: KT-5 (Wilms), KT-14 (rhabdoid), Rh28 (rhabdomyosarcoma), and OS-1 (osteosarcoma)	Variable growth delay; most effective in rhabdomyosarcoma	Twice daily for 6 d/wk for 6 consecutive wk to end point (6 wk)	KT-5, 88%; KT-14, 83%; Rh28, 0% (regrowth); OS-1, 97%
Awasthi et al,^25^ 2012	Pancreas (PDAC)	Modest growth delay	25 mg/kg 5 times weekly to end point (12 d)	PDAC, 80%
Lee et al,^26^ 2013	Non-small cell lung cancer (H292)	Ineffective	50 mg/kg/d from day 0 to day 11	H292, 0%
Halvorson et al,^27^ 2015	Glioma, genetically engineered mouse model	Ineffective	50 mg/kg/d for 21 d	No increase n survival
Carboni et al,^16^ 2009	Colon carcinoma (GEO)	Modest growth delay	25 mg/kg twice daily for 17 d; a list of other xenograft results is reported	GEO, 25%
**CP-751, 871 (figitu-mumab)**				
Cohen et al,^28^ 2005	Colon (colon 205); breast (MCF7)	Modest growth delay	Single dose (antibody)	COLO 205, 56%; MCF7, 20%
Iwasa et al,^29^ 2009	Non–small cell lung (H460 and H1299)	Negligible effect	Single dose (antibody)	H460, 22%; H1299, 17%
Chakraborty et al,^30^ 2015	Breast cancer (BT474 and MCF7)	Ineffective	Weekly for 8 wk	BT474 0%; MCF7 0%
**IMCA12 (cixutu-mumab)**				
Barnes et al,^31^ 2007	Head and neck cancer (TU159)	Cytostasis	Highly variable growth patterns between individual mice with some showing no response	TU159, 100%
Lu et al,^32^ 2005	Pancreatic and colon cancers (BxPC3 & HT29)	Growth delay	Twice weekly for 6 wk	BxPC3, 63%; HT29, 57%
Tonra et al,^33^ 2009	Colon carcinoma (HCT-8 &HT29-LP)	Modest growth delay	3 times weekly to end point	HCT-8, 15%; HT29-LP, 33%
**SCH717454 (robatu-mumab)**				
Wang et al,^34^ 2010	Pediatric tumors: (SK-N-FI neuroblastoma, SJSA-1 osteosarcoma, RD rhabdomyosarcoma)	Tumor regression	Treatments started in unestablished tumors	SK-N-F1, 100%; SJSA-1, 49%; RD, 57%
Kolb et al,^35^ 2008	Pediatric tumor xenografts: (EW5 and CHLA-258, Ewing sarcomas; NB-SD neuroblastoma; OS-1 and OS9, osteosarcomas)	CHLA: regression and regrowth; NB-SD: regression and regrowth	Individual tumor growth curves presented with regrowth after initial inhibition	EW5, 100%; CHLA-258, 0%; NB-SD, 0%; OS-1, 100%; OS-9, 100%
**MK0646 (dalotuzumab)**				
Fagan et al,^36^ 2012	Breast cancer (MCF7L)	Modest growth delay with regrowth	Twice weekly to end point	MCF-7L 0%
Di Cosimo et al,^37^ 2015	Lung adenocarcinoma LXFA629	Left palpable tumors after growth inhibition	Once weekly until 28-d end point (reported TGI 70%)	LXFA629 67%
Lamhamedi-Cherradi et al,^38^ 2016	Ewing sarcoma (EW5 and TC71)	Ineffective as a single agent	Growth delay with regrowth	EW5 0%; TC71 0%
**MM141 (istiratumab)**				
Fitzgerald et al,^39^ 2014	Pancreas (BxPC-3)	Growth delay	Bispecific antibody to IGF-1R and ERBB3	BxPC-3 97%
Camblin et al,^40^ 2018	Pancreas (CFPAC-1, HPAF-11)	Initial growth delay and regrowth	Every 3 d to end point	CFPAC-1 98%; HPAF-11 0%
**OSI906 (linsitinib) (dual IGF-1R and InsR antagonist)**				
Pitts et al,^41^ 2010	Colon (CUCRC007 and CUCRC026)	Growth delay reported 50% CUCRC007) and approximately 75% CUCRC026)	Once daily until end point (25 d)	CUCRC007 61%; CUCRC026 92%
Zeng et al,^42^ 2012	Breast (LCC6)	Modest growth delay	Daily to end point (30 d)	LCC6 56%
Kuhn et al,^43^ 2012	Multiple myeloma (8226.BR)	No growth delay	Stimulation of growth at 20 mg/kg twice weekly	8226.BR −44%
Flanigan et al,^44^ 2010	Colon (HCT15 and CUCRC006)	Growth delay (HCT15) or no effect (CUCRC006)	Dosing to end point (20 or 62 d)	HCT15 48%; CUCRC006 7%
Kim et al,^45^ 2012	Lung (H226B–K-Ras)	No effect	Daily to end point (8 d)	H226B-K-ras 0%
Zinn et al,^46^ 2013	Small cell lung cancer (NCI-H187 and PDXs LX 33, LX36)	Modest growth delay	Daily to end point	H187 64%; LX33 39%; LX36 50%
Ma et al,^47^ 2016	Glioblastoma (GBN76 and 39)	Modest growth delay (GBN76) and stimulation of growth (GBN 39)	Daily to end point	GBM76 44%; GBM39 − 53%
Ramcharan et al,^48^ 2015	Melanoma (A375M)	Growth delay	3 times week to end point (approximately 48 d)	A375M 61%
Min et al,^49^ 2015	Non–small cell lung cancer (H1975)	Modest growth delay	Daily to end point	H1975 17%
Murakami et al,^50^ 2016	Ewing sarcoma (PDX)	Cytostasis	Daily to 14 d; end point 21 d	PDOX 80%

Abbreviations: IGF-1R, insulin-like growth factor-1 receptor; %TGI, percentage of tumor growth inhibition.

^a^ Cell lines used for xenografts.

^b^ Calculated as in Carboni et al.^16^ Estimations of %TGI of single-agent IGF-1R inhibitors on a selection of tumor xenograft models as published in the literature.

## Discussion

From 2003 onward, preclinical data generated by competing pharmaceutical and biotechnology companies propelled 16 IGFIR inhibitor candidates into 183 clinical trials against a broad range of cancers and in a wide array of drug combinations. Details of single-agent trials and of combination trials of IGF-1R with other oncology drugs are detailed in eTables 1 and 2 in Supplement 2. None of these IGF-1R inhibitors received approval for use in oncology practice as single agents or in combinations, although 1 inhibitor (Tepezza, teprotumumab; Roche) was subsequently approved for treatment of Graves disease.^15^ More than 12 000 patients were estimated to have entered these futile trials. Used as single agents, the drugs had an acceptable toxicity profile, although some drug combinations were toxic.^51,52^ For example, in the study of ganitumab with hormonal treatment of receptor-positive breast cancer, serious adverse events developed in 25% of the patients in the ganitumab group compared with a placebo, with the most common grade 3 or higher adverse event being neutropenia.^51^

Publications analyzing these trials addressed important issues that could have explained the lack of clinical efficacy of the IGF-1R inhibitors.^53,54,55,56,57,58,59^ They included redundancy within the IGF-1R–stimulated signaling pathways, with compensation of signaling by alternative pathways—an occurrence common to most signaling inhibitors^60^—and the lack of or failure to use predictive biomarkers for the selection of groups of patients who might have benefited from IGF-1R inhibitor therapy. In a 2017 retrospective analysis of failed late-stage clinical trials, across all of oncology, Jardim and colleagues^61^ concluded that the lack of a biomarker-driven strategy was commonly associated with drug attrition. However, Allison,^54^ in reviewing the IGF-1R inhibitor clinical trials, suggested that most used biomarkers. For example, a preclinical study identified potential biomarkers (IRS2 copy number gain, *KRAS* and *BRAF* mutation status) for the treatment of colon cancer^62^ with the IGF-1R inhibitor BMS-754807; its clinical trial in colon cancer (NCT00908024) was nevertheless a failure (eTable 1 in Supplement 2).

The retrospective commentaries on the failure of the clinical trials of IGF-1R inhibitors^53,54,55,56,57,58,59^ did not question the predictive value of preclinical data deemed sufficient to launch investigational studies in humans. Some of us have contributed to critical comments on preclinical models that fail to capture the key features of a number of diseases, including cancer, and of the decision-making that advances drug candidates to clinical trial.^63^ Those criticisms are pertinent to the models used to select IGF-1R inhibitors for clinical trials. Although it is beyond the scope of this study to review in depth all the preclinical data on IGF-1R inhibitors, we have surveyed the published literature reporting the single-drug activity in vivo in xenografts generated from a wide variety of cancers (Table 2). The results present a mixed picture, with half of these studies resulting in less than a 50% tumor growth inhibition. Many of the in vivo studies sampled reported tumor growth inhibition after immediate and then prolonged treatments of tumors implanted as fragments, treatments often administered before growth was fully established. Most studies also made no estimation of tumor regrowth after treatment cessation. The study of ganitumab (AMG479) by Fahrenholtz et al^17^ is an example of a study in which strong regrowth of the VACaP prostate xenograft was observed immediately on cessation of treatment. This suggests that the cytotoxic (apoptosis-inducing) effects of inhibiting IGF-1 signaling observed in vitro were limited in vivo, leaving viable clones capable of regrowth. Modest inhibition of tumor growth (percentage tumor growth inhibition <50%) by IGF-1R inhibitors in a variety of tumor types, together with tumor regrowth in some assays, should have sent warning signals regarding the status of IGF-1R as a validated drug target, a question raised in a 2009 study of a transgenic mouse model of IGF-1R–induced tumors.^64^

In his 2013 review of the clinical failure of IGF-1R inhibitors, Renato Baserga^56^ referred to “the problem of how cancer cures obtained in mice can be transferred to human beings.” There was no evidence of prolonged cures by IGF-1R inhibitors in tumor-bearing mice (Table 2), despite overall preclinical in vivo data having been described as compelling.^11^

Xenograft data considered to be predictive of the successful clinical activity of IGF-1R inhibitors, all of which subsequently failed to show clinical efficacy, can either be considered to invalidate the models themselves (when there was 100% tumor growth inhibition but no clinical activity) or, at the least, the interpretation of them. In 2014, drug researchers at AstraZeneca articulated a 5-dimensional framework that reduced their drug attrition in clinical trials: more rigorous target validation and improved preclinical models were key aspects that required attention.^65^ This study of IGF-1R inhibitors supports that recommendation, as does the recent suggestion by some of us to use more effective and stringent decision tools during preclinical development.^63^

Published estimates of drug development costs generally focus on successful drug registrations, whereas herein we estimate the cost of failure (Figure 1). The 183 IGF-1R trials had cumulative R&D expenses estimated to be greater than $1.63 billion over the period of our survey. Looking at the drug industry, cancer R&D that is failing or destined to fail will currently incur an annual expense that is in the order of $50 billion to $60 billion (eMethods in Supplement 1).

Moser and Verdin^66^ in 2018 questioned the crowded space of oncology trials, with1405 new molecular entities under investigation in 3158 different indications, many of which are me-too programs. Fojo and colleagues^67^ have also criticized the futility of multiple me-too programs of anticancer drug discovery. What drove 16 companies to dash competitively toward clinical trials of IGF-1R inhibitors, all of which failed? In the early years of the 2000s, the success of trastuzumab may have been a force creating strong expectations.^68,69^ It is also possible that company management, with these high expectations, faced with strong competition and the need to fill their drug pipelines, proceeded in a case of herd instinct, as discussed recently.^70^ To quote Borup et al^71^ in an analysis of expectations in scientific research, “behavior is not only based on rational risk-return considerations, but also influenced by expectations and perceptions of other’s behavior.” It will be interesting in the future to analyze the costs and outcomes of the current intense competition in oncology, with many me-too programs.

What might be learned from the failure of the IGF-1R inhibitor programs? We see at least 3 lessons. The first relates to the evidential hurdles that cancer drugs should pass before moving into the clinic; specifically, the predictive validity of the preclinical models and performance standards that drug candidates should meet in those models.^63^ The second relates to technical diversification to avoid overinvestment in some mechanisms (eg, IGF-1R) and underinvestment in others.^66,67^ The third relates to a lack of rigorous analyses of major translational failures.^63^

### Limitations

This study has limitations. The limitations in estimating drug development costs and failure are elaborated in the eMethods in Supplement 1. In 129 of the trials we found of IGF-1R inhibitors, the Evaluate Ltd algorithm (eMethods in Supplement 1) was used to estimate expenditure on these trials. This algorithm could be challenged, although many international pharmaceutical companies use data generated by it. When we lacked data from Evaluate Ltd, we used their mean expenditure per patient per phase of clinical trial. It is not based on real data that were gleaned from company reports and is therefore questionable. Trials performed by academic centers or institutions, such as the US National Cancer Institute, do not have transparent sources to permit estimations of trial expenditure and so had to be estimated.

We have discussed herein clinical trials and preclinical data that date from the first 2 decades of 2000. It is possible that discovery and improved use of biomarkers for IGF-1R inhibitors could reduce these examples of clinical trial failure, although we are not aware of recent data to substantiate this.

We may have overlooked some of the publications of in vivo preclinical data that allowed us to derive the percentage inhibition of tumor growth by IGF-1R inhibitors. It is also likely that more data, especially from pharmaceutical companies, were not published and that only representative data or optimal data on in vivo assays were published. However, we were able to capture data from 35 publications in which a wide range of cancers xenografted in mice were used. We have not analyzed the data from in vitro tests of IGF-1R inhibitors on cell lines as it is likely that the in vivo data were most important in the decision to progress a candidate drug to clinical trial.

## Conclusions

We do not dismiss the challenges of drug discovery in cancer. During the period that IGF-1R inhibitor projects were launched (1999-2009), it was reported that 83% of the claims that cancer biology could be successfully translated into treatments proved futile, and of drugs that offered an overall survival benefit, it was for a mean of only 6 months.^72^ This limited impact of many systemic therapies to improve overall survival from cancer has continued.^73,74^ The biology of cancer is complex and understanding of cancer mechanisms continues to evolve. In the early years of the 2000s, when IGF-1R inhibitor programs were being launched, 6 hallmarks of cancer were identified; in 2022 this had grown to 14.^75^ Our growing understanding of the complexity and heterogeneity of cancer should provide pause for thought about the human and financial resources involved in the enterprise of drug discovery and to where effort and resources may be better focused to reduce cancer mortality.

## Notes

Supplement 1. **eMethods.** Further Details

Supplement 2. **eTable 1.** Calculation of Trial Expenses in $s **eTable 2.** Basket Trials Not in the Evaluate Ltd Database With the Estimation of Trial Expenses for the IGF-1R Inhibitor Arm Only **eTable 3.** Calculations to Establish the Mean Expenses per Patient per Phase

Supplement 3.
Data Sharing Statement
