# Supplementary material for: Costs and Causes of Oncology Drug Attrition With the Example of Insulin-Like Growth Factor-1 Receptor Inhibitors
Source: JAMA Netw Open. 2023 Jul 28;6(7):e2324977. doi: 10.1001/jamanetworkopen.2023.24977 (PMC10383012; doi:10.1001/jamanetworkopen.2023.24977)
Supplement: Supplement 1. — eMethods. Further Details [file jamanetwopen-e2324977-s001.pdf]

Supplementary Online Content

Jentzsch V, Osipenko L, Scannell JW, Hickman JA. Costs and causes of oncology drug attrition with the example of insulin-like growth factor-1 receptor inhibitors. *JAMA Netw Open*. 2023;6(7):e2324977. doi:10.1001/jamanetworkopen.2023.24977

eMethods. Further Details

This supplementary material has been provided by the authors to give readers additional information about their work.

© 2023 Jentzsch V et al. *JAMA Network Open.*

eMethods Further Details

Primer on R&D cost estimates

Biopharmaceutical R&D cost estimates are contentious, not least because they have been politicized to use in the policy debate on drug pricing[1–3]. It sometimes appears that advocacy, not rigor, is the primary analytic objective. So, for example, the drug industry has used "recouping R&D cost" arguments to say that drugs are expensive because R&D is expensive. This argument is poor because it invokes the sunk cost fallacy[a],[1–3]. However, in response to this poor argument, some industry critics seem eager to show that R&D is implausibly cheap, perhaps imagining that this will reduce drug prices[3].

Political polarization aside, much of the disparity in cost estimates can be traced to two sources. The first is the methodological variation. The second is heterogeneity in the sample of R&D projects chosen for analysis. We start by introducing methodological variation, move on to project sampling, and then try to place the cost estimates in our paper within the wide methodological and sampling milieu that exists in the literature.

For readers who want a flavour of the factors that drive of variation in R&D cost estimation, we recommend Schlander et al.[4] as a good starting point.

Methodological heterogeneity in R&D cost estimates

"We find no sense in talking about something unless we specify how we measure it: a definition by the method of measurement is one sure way to avoid talking nonsense" [5]

Standard accounting, of the kind that produces annual income statements, cashflow statements, and balance sheets, is based on standard rules (e.g., US Generally Accepted Accounting Principles or US GAAP). The rules are well suited to some financial analyses but not others.

Accounting statements provide a good picture of simple businesses where income and expenses are matched within a single accounting period. Consider a tobacco farm[b]. Each year, the farmer buys seed, fertilizer, tractor fuel, etc., plants the seed, lets it grow, and then harvests and sells the tobacco. It is therefore relatively easy to use the annual accounts answer the question *"how much did this year's tobacco harvest cost to grow?"*.

Things are different in a forestry business. Each year, the owner buys and plants saplings which will be harvested several decades later. The owner also and harvests and sells trees which are several decades old. Accounting rules provide little guidance on how to allocate the cost of labour, fuel, machinery, etc., that were incurred over many years to a particular cohort of trees. The annual accounts do not tell us how much it cost to grow this year's timber. They are even less help in determining the cost of raising an individual tree.

In this respect, pharmaceutical R&D resembles forestry and not tobacco farming. Researchers who want to know how much a set of drugs cost to discover and develop are forced to adopt non-standard methods. They can apply such non-standard methods to public accounting data that were never designed to help with product-specific cost estimates[6–9]. Alternatively, they can apply a different set of non-standard methods to project-level company data that is more convenient to use but which is not disclosed in public accounts[10,11]. And even then, sensible methods that are competently applied will vary in how the deal with the cost of failed projects, the cost of capital, in the inclusion or exclusion of a range of other costs, etc.

Cost of failure and the cost of capital

[a] An accurate framing is that high drug prices incentivise private sector R&D investment, which is not the same thing as allowing the private sector to "recoup" past investment. But this framing is, arguably, less palatable for lobbying and public relations purposes [2,3].
[b] With thanks to Richard Evans for this example

© 2023 Jentzsch V et al. *JAMA Network Open.*

Two very important methodological choices are illustrated in Table 1. The data are based on figures in a classic paper on pharmaceutical R&D productivity published by Paul et al.[12] The table shows an archetypical "big pharma" cost structure in the years running up to the paper's publication in 2012. It represents the "average" activity that leads to a single drug approval, with 24 starting projects ("Target to hit") whittled down over 13.5 years to a single FDA approval. While one should not give undue weight to these specific figures, the phase-specific costs and timings are regarded as plausible enough to serve as default values, or placeholders, in certain biotech industry forecasts in the absence of more detailed information[13].

Table 1

	Number of projects (WIP)	Cash cost per project ($m)	NPV per project ($m)	Cash cost per launch ($m)	NPV per launch ($m)	Time cost of money	Years prior to launch
Target to hit	24.3	-1	-4	-24	-99	409%	13.5
Hit to lead	19.4	-3	-9	-49	-179	369%	12.5
Lead optimsation	14.6	-10	-32	-146	-460	315%	11.0
Preclinical development	12.4	-5	-13	-62	-159	256%	9.0
Phase 1	8.6	-15	-35	-129	-297	230%	8.0
Phase 2	4.6	-40	-79	-184	-363	197%	6.5
Phase 3	1.6	-150	-228	-240	-364	152%	4.0
Submission to launch	1.1	-40	-47	-44	-51	117%	1.5
Approval event	1.0	0	0	0	0	0	0
**Total ($m)**		**-264**	**-445**	**-878**	**-1973**		
Of which clinical trials ($m)		-205	-341	-553	-1024		

The cash cost per project in Table 1 can be thought of as the average amount of cash that one would need to run a single programme through a given phase of discovery or development. The phases vary in length. In reality, a clinical phase may involve a single trial or many (see below). A clinical phase also includes non-trial costs (e.g., manufacturing the experimental medicine, data analysis for regulatory submissions, etc.).

Table 1 shows an archetypical portfolio spread over time, eventually funnelling down to a single approval. A given year's R&D expense in the company accounts would approximate the sum of cash costs of all the projects running that year. The R&D expense would only approximate these cash costs because accrual accounting (e.g., US GAAP) and cash accounting are different. Accrual accounts are reconciled with cash accounts via items such as amortisation, depreciation, CAPEX, etc. Furthermore, Paul et al., excluded some items that are recorded as R&D expenses, such as early-stage drug discovery, post-approval R&D, and perhaps some corporate overheads that would be allocated to R&D.

There are four "Total" R&D costs per approved drug at the foot of Table 1. They vary by a factor of 7.5, from $264m to $1973m. The per-project totals ($264m and $445m) are measures of the average cost of the R&D on the single approved drug. The per-launch costs ($878m and $1973m) include the cost of the failed projects.

The "cash cost" totals ($264m and $878m) approximate the costs that would have shown up in annual accounts over the years. The NPV totals ($445m and $1973m) adjust for the time cost of money, in this case assuming that the annual cost of capital is 11%. This inflates historic costs versus the time of launch. Investors (e.g., biotech venture capitalists, drug industry shareholders, etc.) disagree on the precise figure, but the cost of capital is a major consideration in decisions to allocate money to R&D.

So, which is the "correct" cost?

There is no clear answer[4]. Different cost estimates apply to different things and serve different purposes. The $264m figure could be relevant to someone interested in the operational budget of a specific R&D project (or to someone whose policy objective was to argue for low drug prices). The $878 figure might be useful for a financial analyst who is trying to calculate an R&D-adjusted return on equity measure to compare the drug industry with other industries. The $1973m figure is more relevant to an investor deciding whether to provide capital to fund the R&D portfolio (or to a drug industry lobbyist arguing for high prices).

© 2023 Jentzsch V et al. *JAMA Network Open.*

A wide range of other cost allocation choices

Over and above the cost of failure and the cost of capital, different R&D cost estimates vary widely in terms of the other costs they chose to include and exclude [4].

The figures in Table 1[12] exclude early-stage drug discovery activities that come before the "target to hit" stage. They exclude post launch R&D costs. They also exclude some "overheads" such as the salaries of staff who support R&D but who are not directly engaged in R&D.

Consider trials that are run across one or more academic centres. There are large variations in the "overheads" or indirect costs that different centres add to the direct cost they estimate for performing the clinical work[14,15]. If the trial is funded by a philanthropic body, the cost will sometimes exclude the University's "overheads". If the funder is a for-profit entity, then "overheads" can double the cost. Overheads are charged on work funded by bodies like the NIH, but even here rates vary between institutions[15,16].

One can find in the literature R&D cost figures that take an explicitly narrow view and consider only clinical trials[17]. Others take a very broad view and compare industry-wide R&D expenses with drug approvals, either lagged[7,8] or unlagged[6,8]. Others fall somewhere in between[10–12].

As an aside, there will also be differences in project cost allocation methods between companies. Were two different large drug companies running two identical R&D projects, one would not necessarily expect them to compute identical project costs.

Sampling error and bias in R&D cost estimates

Even if one eliminates methodological differences, there remain huge differences between the cost of different R&D programmes. Sampling is important[4].

Consider for example, canakinumab, an antibody that binds interleukin-1β. Canakinumab was first approved in CAPS a rare inflammatory disorder. The approval in CAPs depended on two trials conducted in a few tens of patients and lasting no more than 8 months [18]; let's say a total of 40 patient years of drug exposure. More recently, canakinumab was tested as a drug to reduce cardiovascular risk [19]. The trial planned to enrol 17,000 patients and track them over 5 years. In the end, it enrolled over 10,000 who – as of results publication in 2017 – had been tracked for a median of 3.7 years; let's say 40,000 patient years of drug exposure.

The literature, in our view, tends to under-emphasize this kind of heterogeneity because it typically reports average costs, sometimes by indication or development phase (e.g., Table 1, Refs [12,13]) . The author's experience of the drug and biotech industries suggests that small commercial phase III programmes in a rare disease may have cash cost in the low to mid tens of millions of dollars, while the biggest and longest cardiovascular trials (e.g., involving multi-year hard outcome trials in tens of thousands of patients) cost several hundreds of millions of dollars.

Furthermore, very successful drugs can attract huge R&D investment after their first approval in their first indication. Pembrolizumab, for example, was first approved for use in advanced melanoma in 2014. As of October 2022, it had been approved in 18 different oncology indications and a brief search of clinicaltrials.gov revealed over 1200 industry sponsored trials involving the drug.

If, as appears to be the case, R&D costs are highly variable and highly skewed (i.e., a tail of extremely costly programmes such as those involving pembrolizumab in oncology or canakinumab in cardiovascular disease, large cardiovascular programmes, large Alzheimer's programmes, etc.), sampling error is likely large even when cost estimates consider a large number of programmes. This is an intrinsic feature of some highly skewed distributions.

A second sampling problem in some published R&D costs estimates is that of survivor bias which, in turn, is likely to artificially deflate R&D cost estimates. This occurs when one bases analyses on companies that have brought drugs to market and excludes from the analysis the R&D costs of companies that have not brought drugs to market. For an example of an analysis that, in our view, risks this kind of bias, see Prasad and Mailankody (2017)[9].

© 2023 Jentzsch V et al. *JAMA Network Open.*

- **Locating our IGF1R estimates within the methodological and sampling milieu**

The most obvious difference between our IGF1R estimates and those in the bulk of the literature is that the literature focuses on the cost of success, generally R&D costs per approved drug[2,4]. This requires the sample to include at least one approved drug. We have focused on the cost of failure.

Turning to methodology, our measure, based on proprietary commercial data from Evaluate, is an accounting-based measure. It does not include the cost of capital. Our estimates map roughly onto figures that would be reported within the R&D expense line in quarterly and annual company accounts. They correspond roughly to the cash cost columns in Table 1. Therefore, we generally call them "expenses" in the main paper.

Were we to include the time cost of money at 11%, discounted to the target approval year, and given that we focused on clinical development, one would multiply our R&D expense estimate by roughly 2x to calculate their Net Present Value (NPV, see the "Time cost of money" column in Table 1, applied to Phases 1, 2, and 3).

Our measure excludes R&D expenses incurred before Phase I and after Phase III trials. The ratio of discovery and preclinical costs to clinical development costs depends, of course, on how one deals with the cost of failure and the time cost of money. If one assumes the IGF1R agents resembled Table 1 up to Phase III, then discovery and preclinical costs would add 10% to 15% to the expense estimate or 40% to 50% to an NPV estimate.

We make two other points. First, our measure will include some non-trial expenses that are incurred while trials are running. However, companies vary in how they allocate the drug-level expenses that they disclose in their 10-K filings (e.g., some will allocate more overheads, some less). Second, Evaluate's benchmarks are based on historic 10-K filings (and other public sources) but are not inflation adjusted. If we assumed that the benchmarks are based on data that are 7 years old and applied a 5% annual R&D cost inflation figure (probably low), it would inflate our expense estimates by around 40%.

If we had chosen to include the cost of capital, preclinical and discovery costs, and inflation, our aggregate R&D expense estimate of around $2 billion for the IGF1R inhibitors would rise to around $6.5 billion and perhaps more. This illustrates our view that headline figures associated with R&D cost estimates are very hard to interpret without detailed consideration of the methods that have been used to derive them[5].

Rough estimate of the annual R&D expense of failed oncology drug R&D at an industry level

The global biopharmaceutical industry has aggregate annual R&D expenses of around $200 billion [20]. Oncology drugs make up around 40% of all compounds in clinical development[21] and, we assume, a similar proportion of preclinical pipeline projects. Given multiple indications in oncology and the relatively high cost of cancer trials, we assume cancer projects incur 45% of all R&D expenses. For archetypical R&D portfolios, 70% of expenses are associated with failed projects (see $878 million for R&D costs including failure and $264 million for R&D costs excluding failure in Table 1). Of course, oncology attrition is relatively high so 70% may be an under-estimate of the cost of failure in this case. However, if we apply the 70% cost of failure to the 45% of the R&D activity that is oncology related to $200 billion annual R&D expense, then failed oncology projects incur and annual R&D expense in the $50 to $60 billion range.

© 2023 Jentzsch V et al. *JAMA Network Open.*

eReferences

1. Big pharma says drug prices reflect R&D cost. Researchers call BS | Ars Technica. https://arstechnica.com/science/2022/10/big-pharma-says-drug-prices-reflect-rd-cost-researchers-call-bs/.
2. Wouters, O. J., Berenbrok, L. A., He, M., Li, Y. & Hernandez, I. Association of Research and Development Investments With Treatment Costs for New Drugs Approved From 2009 to 2018. *JAMA Netw. Open* **5**, e2218623 (2022).
3. Scannell, J. *Four Reasons Drugs are Expensive of Which Two are False*. 22 https://www.innogen.ac.uk/sites/default/files/2019-08/Innogen-Working-Paper-114---Final-1.pdf (2015).
4. Schlander, M., Hernandez-Villafuerte, K., Cheng, C.-Y., Mestre-Ferrandiz, J. & Baumann, M. How Much Does It Cost to Research and Develop a New Drug? A Systematic Review and Assessment. *PharmacoEconomics* **39**, 1243–1269 (2021).
5. Bondi, H. *Relativity and Common Sense*. (Heinemann, 1965).
6. Herper, M. The Truly Staggering Cost Of Inventing New Drugs. *Forbes* https://www.forbes.com/sites/matthewherper/2012/02/10/the-truly-staggering-cost-of-inventing-new-drugs/.
7. Scannell, J. W., Hinds, S. & Evans, R. Financial Returns on R&D: Looking Back at History, Looking Forward to Adaptive Licensing. *Rev. Recent Clin. Trials* **10**, 28–43 (2015).
8. Scannell, J., Blanckley, A., Boldon, H. & Warrington, B. Diagnosing the decline in pharmaceutical R&D efficiency. *Nat. Rev. Drug Discov.* **11**, 191–200 (2012).
9. Prasad, V. & Mailankody, S. Research and Development Spending to Bring a Single Cancer Drug to Market and Revenues After Approval. *JAMA Intern. Med.* **177**, 1569–1575 (2017).
10. DiMasi, J. A., Hansen, R. W., Grabowski, H. G. & Lasagna, L. Cost of innovation in the pharmaceutical industry. *J. Health Econ.* **10**, 107–142 (1991).
11. DiMasi, J. A., Grabowski, H. G. & Hansen, R. W. Innovation in the pharmaceutical industry: New estimates of R&D costs. *J. Health Econ.* **47**, 20–33 (2016).
12. Paul, S. M. *et al.* How to improve R&D productivity: the pharmaceutical industry’s grand challenge. *Nat. Rev. Drug Discov.* **9**, 203–214 (2010).
13. David, F., Robey, S., Matthews, A. & MD, D. S. *The Pharmagellan Guide to Biotech Forecasting and Valuation*. (Pharmagellan, 2017).

© 2023 Jentzsch V et al. *JAMA Network Open.*

14. Hind, D. *et al.* Comparative costs and activity from a sample of UK clinical trials units. *Trials* **18**, 203 (2017).

15. Johnston, S. C., Desmond-Hellmann, S., Hauser, S., Vermillion, E. & Mia, N. Predictors of Negotiated NIH Indirect Rates at US Institutions. *PLOS ONE* **10**, e0121273 (2015).

16. Kaiser, J. NIH overhead plan draws fire. *Science* **356**, 893–893 (2017).

17. Sertkaya, A., Wong, H.-H., Jessup, A. & Beleche, T. Key cost drivers of pharmaceutical clinical trials in the United States. *Clin. Trials* **13**, 117–126 (2016).

18. ILARIS® (canakinumab) for injection, for subcutaneous use.

19. Ridker, P. M. *et al.* Antiinflammatory Therapy with Canakinumab for Atherosclerotic Disease. *N. Engl. J. Med.* **377**, 1119–1131 (2017).

20. *The Pharmaceutical Industry and Global Health: Facts and Figures 2021*. https://www.ifpma.org/wp-content/uploads/2021/04/IFPMA-Facts-And-Figures-2021.pdf (2021).

21. Moser, J. & Verdin, P. Burgeoning oncology pipeline raises questions about sustainability. *Nat. Rev. Drug Discov.* **17**, 698–699 (2018).

© 2023 Jentzsch V et al. *JAMA Network Open.*
