# Supplementary material for: Costs and Causes of Oncology Drug Attrition With the Example of Insulin-Like Growth Factor-1 Receptor Inhibitors
Source: JAMA Netw Open. 2023 Jul 28;6(7):e2324977. doi: 10.1001/jamanetworkopen.2023.24977 (PMC10383012; doi:10.1001/jamanetworkopen.2023.24977)
Supplement: Supplement 3. — Data Sharing Statement [file jamanetwopen-e2324977-s003.pdf]

Data Sharing Statement

Jentzsch. Costs and Causes of Oncology Drug Attrition With the Example of Insulin-Like Growth Factor-1 Receptor Inhibitors. *JAMA Netw Open*. Published July 28, 2023. doi:10.1001/jamanetworkopen.2023.24977

Data
Data available: Yes
Data types: Deidentified participant data
How to access data: Supplementary Excel eTables S1 and S2
When available: With publication

Supporting Documents
Document types: Other (please specify)
Additional Information: Discussion of the costs of drug development
How to access documents: Supplementary discussion
When available: With publication

Additional Information
Who can access the data: anyone who asks for it
Types of analyses: Expenses associated with clinical trials
Mechanisms of data availability: Online Excel files
Any additional restrictions: None
